# The Cognitive Daisy (COG-D) for improving care for residents with dementia in care homes: protocol of a feasibility RCT

**DOI:** 10.1186/s40814-023-01256-8

**Published:** 2023-03-03

**Authors:** Petra M. J. Pollux, Claire Surr, Judith Cohen, Chao Huang, Emma Wolverson, Pauline Mountain, Rebecca Turner, Emma Hawkesford-Webb, Bethany Winter, John M. Hudson

**Affiliations:** 1 University of Lincoln, Campus Way, Lincoln, LN6 7TS UK; 2 City Campus University of Leeds-Beckett, Calverley, 521, Leeds, LS18 4RQ UK; 3 Hull Health Trials Unit, Hull York Medical School, Allam Medical Building, University of Hull, Hull, HU6 7RX UK; 4 Hull York Medical School, Allam Medical Building, University of Hull, Hull, HU6 7RX UK; 5 Faculty of Health Sciences, University of Hull, Cottingham Road, Hull, HU6 7RX UK; 6 5 Foster Close, Timberland, LN4 3SE UK

**Keywords:** Dementia, Person-centred care, Cognition-focussed assessment and care-planning, Care homes

## Abstract

**Background:**

Cognitive problems associated with dementia affect a large proportion of older adults living in residential care. Knowledge of cognitive impairments is important for providing person-centred care (PCC). The impact of specific cognitive impairments on residents’ needs is often overlooked in dementia training and information about residents’ individual cognitive profiles are frequently underspecified in care-plans, potentially undermining the delivery of PCC. This can lead to reduced resident quality of life and increased distressed behaviours—a major cause of staff stress and burnout. The COG-D package was developed to fill this gap. Daisies provide a visual representation of a resident’s individual cognitive strengths and weaknesses in a colourful flower (Daisy) representing five cognitive domains. By viewing a resident’s Daisy, care-staff can flexibly adjust in-the-moment care-decisions and can consult Daisies in care-plans for longer-term planning. The primary aim of this study is to assess the feasibility of implementing the COG-D package in residential care homes for older adults.

**Methods/design:**

This 24-month feasibility cluster randomized controlled trial involves a 6-month intervention of the use of Cognitive Daisies in 8–10 residential care homes for older adults after training of care staff on the use of Cognitive Daisies in daily care (basic training) and on conducting the COG-D assessments with residents (advanced training). The key feasibility outcomes include % residents recruited, % COG-D assessments completed, and % staff completing the training. Candidate outcome measures for residents and staff will be obtained at baseline, and at 6 and 9 months post-randomization. COG-D assessments of residents will be repeated 6 months after the first assessment. A process evaluation will assess intervention implementation and barriers and facilitators to this through care-plan audits, interviews and focus groups with staff, residents, and relatives. Feasibility outcomes will be analysed against progression criteria to a full trial.

**Discussion:**

The results of this study will provide important information about the feasibility of using COG-D in care homes and will inform the design of a future large-scale cluster RCT to assess the effectiveness and cost-effectiveness of the COG-D intervention in care homes.

**Trial registration:**

This trial was registered on 28/09/2022 (ISRCTN15208844) and is currently open to recruitment.

## Background

Around 400,000 adults live in residential care [1], at least 70% have dementia [2] and of those 78% experience distress behaviours staff find challenging to support [3]. These have widespread impacts on resident and staff quality of life [4] and present significant financial costs to the NHS and social care [5]. Distress behaviours such as agitation, disinhibition, and aggression, cause significant suffering for the person with dementia, distress for those around them and burden and stress for care-staff. Their causes are multifactorial, including staff not always having adequate understanding of the impact of cognitive impairments on day-to-day functioning [6]. Cognitive impairments in dementia are complex as they vary as a function of dementia subtype [7] and premorbid cognitive status [8] and may fluctuate [9] and worsen with disease progression [10]. The importance of knowledge about a resident’s unique cognitive profile is acknowledged in best-practice models of Person-Centred Care (PCC) [11] and are considered core knowledge for social care-staff [12]. However, providing an in-depth understanding of the cognitive difficulties experienced in dementia is an area frequently overlooked in dementia training [13]. In practice, the available information about a resident’s cognitive problems is often underspecified (e.g., diagnosis only), not readily available to care-staff, incomplete or out of date [14]. Consequently, care-staff can have difficulty identifying needs that arise from specific cognitive problems and to adjust their care practices accordingly. This can lead to excess disability, seen in greater dependence in activities of daily living that a resident is potentially capable of.

**Fig. 1:**
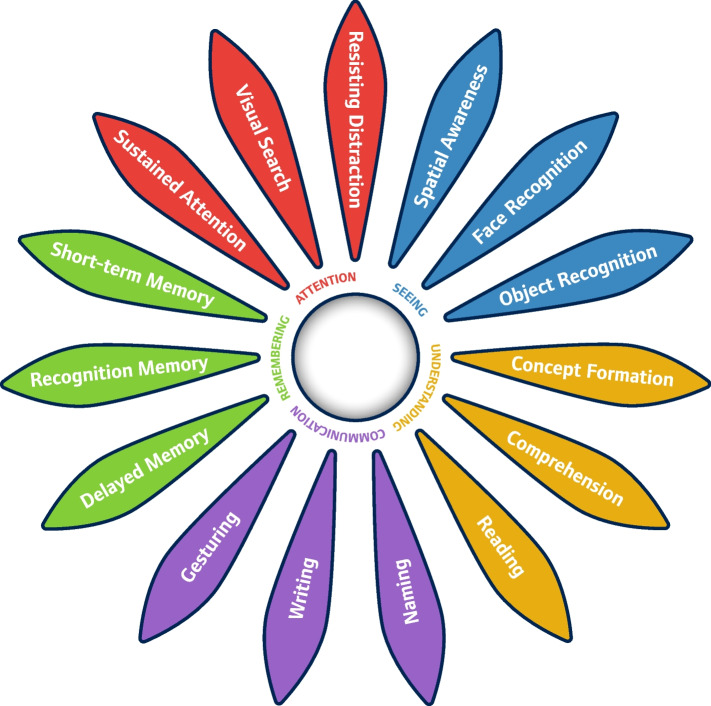
The Cognitive Daisy

The Cognitive Daisy (COG-D, Fig. 1) intervention [15] includes an assessment of cognitive abilities and provides a visual summary of the cognitive profile of a person with dementia to inform care-planning and practice. The COG-D visualizes a person’s cognitive strengths and limitations across five cognitive domains (visuo-spatial perception, comprehension, communication, memory, and attention) in the form of a Cognitive Daisy with 15 petals, each corresponding to one of the COG-D assessment areas. A coloured petal indicates that the person is likely to experience problems in this cognitive domain, whereas a white petal means they are not likely to show any difficulties in the corresponding area. The COG-D is not a diagnostic tool but assesses whether (as opposed to ‘how well’) a resident can perform a specific task (e.g., the ability to write a few words can be sufficient for effective communication of needs). The cut-off scores for coloured petals are based on normative data obtained from older adults living independently in the community who had no diagnosed cognitive impairments. COG-D assessment scores have high internal consistency and concordance with the 6-item cognitive impairment task [16] in normative data collected from healthy older adults [10].

The COG-D intervention package includes (1) staff training, consisting of (i) basic training for all care-staff on cognition in dementia and how Cognitive Daisies are used in daily practice, and (ii) advanced training for senior care-staff on conducting COG-D assessments; (2) completion of resident COG-D assessments and creation of individual Cognitive Daisies including specific care recommendations (6–8 common functional problems and suggested solutions automatically generated by a user-friendly application or via a printed ‘Petal-By-Petal Guide’). (3) Integration of COG-D in care-plans and display of the Daisies in useful locations (e.g., residents’ rooms). (4) Re-assessment if cognitive changes are noticeable in several domains, or on a 6-monthly basis.

**Fig. 2:**
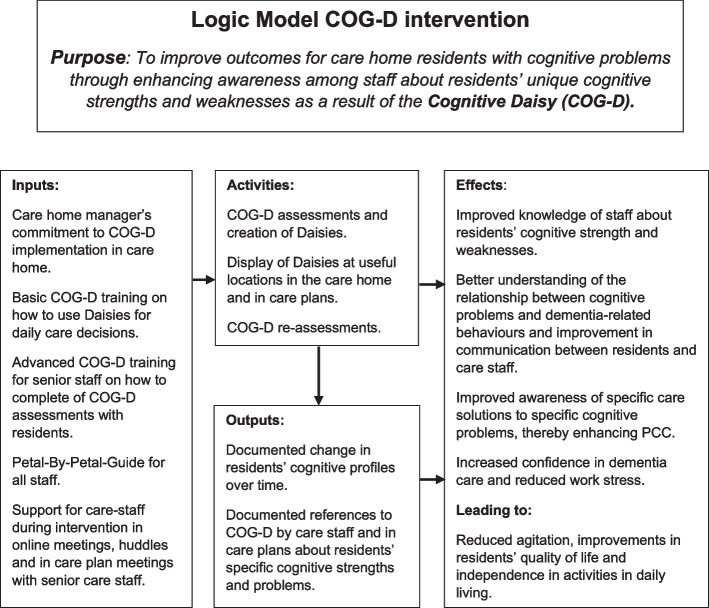
Logic model for the COG-D intervention

In accordance with our Logic Model (see Fig. 2), implementation of the COG-D intervention is expected to (i) help care-staff to identify needs that arise from specific cognitive problems, (ii) enhance their understanding of how they could respond to these needs, (iii) provide practical approaches to meeting cognitive needs (iv) leading to a reduction in distress behaviours and improved quality of life for residents, increased sense of competence and reduced stress for care home staff and cost savings to the health and social care system.

There is sparse literature on care home staff knowledge about cognition in dementia and interventions to support cognition-focussed assessment and care-planning, indicating that this is an area where there is a need for interventions to support the social care workforce. In development work in care homes, COG-D assessments were conducted for 33 residents across 6 care homes [10]. Care-staff (*n* = 27) rated the Daisies as improving their understanding of residents’ cognitive problems, found it easier to remember the specific problems of each resident and rated the Daisies as highly useful for delivery of PCC. Before the COG-D intervention can be rolled-out more widely, a more rigorous evaluation of the effectiveness and cost effectiveness of the intervention for residents is required. We first need to conduct a feasibility study to test some uncertainties about the study design, implementation in a larger number of care homes, and patient recruitment and retention. If the protocol for the study is feasible, the results will inform the design for a large-scale multi-centre, definitive cluster RCT, including choice of outcome measure, sample size, and strategies to respond to any identified barriers and facilitators of the study procedures, implementation, and training in care homes.

## Objectives

### Primary objective

To investigate the feasibility of conducting a future large-scale cluster RCT (cRCT) to assess the effectiveness and cost-effectiveness of the Cognitive Daisy (COG-D) intervention in care homes. Feasibility of a future cRCT will be investigated with regard to the following areas of uncertainty: (1) recruitment and retention of care homes, residents and care-staff in both intervention and control conditions, (2) adherence to COG-D intervention protocol, (3) acceptability and data completion rates of the candidate outcome measures, (4) estimates of effect sizes of proposed outcome measures to establish primary endpoint and sample size for the future CRT, (5) ability to collect health economic data required to undertake a cost-effectiveness analysis in the definitive trial.

### Secondary objectives

1. To investigate any signal of efficacy of the Cognitive Daisy (COG-D) on a range of outcome measures for residents and staff.
2. To explore implementation and pathways of impact of the COG-D in care homes in a process evaluation.

### Trial design

A cluster-randomized controlled feasibility trial (cRCT) of usual care plus the Cognitive Daisy intervention (COG-D) compared to usual care (control), with two parallel arms and a 1:1 allocation ratio.

## Methods

### Study setting

Eight to 10 care homes for older adult care (on average 10–13 residents per care home) will be recruited in one region in the north-east of England from care home networks and organizations with whom the trial team have an existing relationship and who are not currently using COG-D. Selected care homes will vary in terms of type (residential/nursing) to include residents at different stages of dementia, and will not have been involved in the COG-D pilot study [10].

### Eligibility criteria (Table 1)

**Table 1:** Eligibility criteria for care homes, care home residents, and care staff

Inclusion criteria:	Exclusion criteria:
*Care homes*	
• Residential or nursing home for older adults	• Have already used COG-D
• Provides care to people with dementia • Has minimum of 20 beds to facilitate recruitment of a sufficient number of residents in each care home	• Is subject to Care Quality Commission enforcement notices Local Authority quality concerns (to reduce care home burden) • Is involved in another complex intervention trial at the time of recruitment
*Residents—all*	
• Consenting to take part (personal or via consultee)	• Receiving end of life care
*Residents—taking part in COG-D assessment*	
• Can communicate well enough in English to not require an interpreter	
• Vision/hearing (with correction) adequate to participate in COG-D assessment	
• Deemed well enough by staff/researcher to complete the assessment by a member of staff or the assessor	
*Care home staff*	
• Permanent, contracted, agency, or bank member of staff at time of data collection	• Acting as a nominated consultee for any residents participating in the trial
• Provide informed consent	
• Have sufficient proficiency in English to contribute to the data collection required for the research	

### COG-D intervention

The COG-D intervention will be commenced in all intervention arm care homes immediately post-randomization and consists of the following two phases: Phase 1: staff training and COG-D assessments of residents’ abilities, phase 2: COG-D use in daily practice (6 months) and COG-D re-assessment of residents’ abilities.

#### Phase 1

##### Staff training

Basic COG-D Staff Training (~ 45 min): aimed at all care-staff who will use the Cognitive Daisy in daily care. The training introduces the Cognitive Daisy, describes the five cognitive domains represented in the coloured petals and the different functions association with each petal with reference to the Petal-By-Petal guide. To accommodate care home preferences and the importance of flexibility in terms of mode of delivery (e.g., temporary care home closure), this training can be completed either individually (online), in groups of staff from single or multiple care homes using videoconferencing, or in single care home groups face-to-face. This feasibility study will explore the use of these different modes of delivery and the reasons thereof.

Advanced COG-D Staff Training (~ 3 h): aimed at senior staff who will be involved in the COG-D assessment of residents. This practice-based training introduces staff to the COG-D assessment process, the individual assessment items, how to create Daisies and how to use the Daisies and the Petal-By-Petal guide to update a resident’s care-plan. This training can be completed in groups of staff from single or multiple care homes, either in person or in videoconferencing sessions.

##### COG-D assessments of residents

The COG-D assessments will be completed by a researcher and shadowed by senior staff who completed the Advanced COG-D Staff Training. The assessment tool COG-DAA constitutes 16 tasks, variants of which are routinely used for assessment of dementia and other neurological disorders. Assessments are conducted using the assessment booklet (A6 size), which allows assessment in a comfortable environment. A detailed COG-D assessment script is used with verbatim instructions for each test. The order of the tests allows for breaks without affecting performance. The test will be introduced as ‘puzzles’ or ‘activities’ to reduce any negative associations or performance anxiety. All materials are presented in a large font and line drawings are avoided (for instance, photographs of real objects are used for object naming and object recognition). A version for non-verbal responses is included to accommodate people with problems with speech production (COG-D SSP). Word lists for the different tests were carefully balanced in terms of age of acquisition, word-length, word-frequency, semantic category, and concreteness [15].

If the score for a test is below the cut-off score, the petal associated with the specific test is coloured. If the test cannot be completed, the petal will be grey. Daisies can be created in different ways to accommodate different uses. Assessors can choose to use a sticker system to create the Daisy together with the resident, or to create a digital version (using a web-based application) for inclusion in care plans (or both). For both methods, the Daisies can be produced without labels (for display) or with labels (e.g., for care plans). The digital version includes the pages of the Petal-By-Petal guide for the coloured petals. The study will explore how the different methods are used in the care homes.

COG-D re-assessment is conditional for the usefulness of COG-D. A full re-assessment is recommended if cognitive changes are noticeable in several domains or following a significant change in a resident’s health status. Individual skills can be re-assessed if changes are observed for specific cognitive abilities. Social context or time-of-day fluctuations in performance may occur; re-assessment of an individual test is recommended if the result deviates consistently from observations of care-staff or family/relatives. In the present study, COG-D re-assessment is only conducted after the COG-D use in Daily Practice intervention period.

#### Phase 2

##### Use of COG-D in daily care practice

The Daisies without labels will be placed in locations where they are most useful (e.g., in residents’ rooms) and Daisies with labels are included in the care-plans. Recruited staff will be provided with the Petal-By-Petal Guide. Larger posters of the Cognitive Daisy (not resident-specific version) will be displayed in dining areas and in communal spaces where scheduled activities take place.

To support staff and to monitor use of resident Daisies, a range of activities will be arranged during this phase of the intervention: (1) weekly guided ‘huddles’ [17] and fortnightly virtual COG-D drop-in sessions for staff to ask questions about the use of Cognitive Daisies, share good practice and to monitor use; (2) care plan review meetings (after the initial and the second COG-D assessment period) to discuss how Daisies can be used to update care-plans; (3) monthly COG-D Assessor drop-in sessions (senior staff) to ask questions and for sharing of good practice; (4) monthly newsletters to update intervention arm care homes about the progress of the COG-D project and to summarize suggestions on the use of COG-D in practice, based on the huddle and drop-in session discussions. Control homes will receive the newsletter content related to general study progress without any COG-D specific content.

##### COG-D re-assessment of residents

Six months after the first COG-D assessment, residents will be re-assessed on COG-D by researchers and senior staff. Senior staff will be encouraged to lead the assessments and both assessors will score performance independently for concordance rates.

### Intervention fidelity and adherence

Fidelity of the intervention will be optimized by using the same researchers for providing the Advanced Training in all intervention care homes and the same training materials for face-to-face and online training sessions for the Basic Training. COG-D assessors use a detailed COG-D assessment script with verbatim instructions for each test and detailed scoring instructions are provided. If COG-D re-assessments are led by senior staff, scoring is conducted independently by both assessors to ensure consistency. Fidelity and adherence will be monitored during the huddles and drop-in sessions where staff can ask questions and share good practice under supervision of the researchers. Fidelity and adherence will further be explored during the focus groups with staff during the process evaluation phase to inform the staff training and monitoring processes for the future trial.

### Usual care

Usual care is defined as normal care delivered within the setting, which will continue in both arms. No restrictions will be imposed on current practices or on homes undertaking additional development or training as part of usual care, except for control arm homes being required not to implement COG-D during their trial involvement period.

### Outcomes

Primary outcome of this study are the feasibility outcomes, including recruitment and retention rate, acceptability, fidelity and adherence to COG-D intervention protocol, acceptability and data completion rates, and feasibility of health economic assessment. Key feasibility criteria a presented in Table 2. Secondary outcomes are the candidate outcomes for a definitive cRCT (see Table 3).

**Table 2:** Key progression criteria

*Key progression criteria*	
Percentage recruited residents	*Green > 60%, amber 40–60%, red < 40%*
Percentage of recruited residents for completion of COG-D and COG-D re-assessment	*Green > 60%, amber 40–60%, red < 39; re-assessment: 70% of these estimates*
Percentage of recruited staff completing basic training	*Green > 60%, amber: 40–60%, red < 40%*
Percentage of recruited senior staff completing advanced training	*Green > 75%, amber 60–74%, red < 60%*

**Table 3:** Candidate outcome measures

Outcome measures	Outcome	Description
*Residents*		
Cohen-Mansfield Agitation Inventory CMAI [23, 24]	Agitation	Lists 29 agitated behaviors. By proxy rating of frequency of occurrence in the previous 2 weeks, ranging from never (1) to several times an hour (7). Scores range from 29 to 203. A high score indicates high levels of agitation.
Quality of Life in late-stage Dementia scale QUALID [25]	Quality of Life	By proxy ratings (1 to 5) of 11 behaviors. Scores range from 11 to 55; low scores indicate higher levels of quality of life.
Bristol Activities of Daily Living Scale BADLS [26]	Independence in daily activities	By proxy rating (1–5) of average ability on 20 activities in the preceding 2 weeks. Scores range from 20 to 100. Low scores indicate higher levels of independence.
EQ-5D-5L: Instrument to describe and value health [27]	Health-related quality of life	By proxy rating (1–5) of health-related quality of life in five domains. Scores range from 20 to 100. A low score indicate a higher level of health-related quality of life.
Functional Assessment Staging of Alzheimer’s disease FAST [20]	Estimate of dementia stage	Functional scale designed to evaluate people at the more moderate-severe stages of dementia. The scale has seven stages, the highest rating (7) indicates ‘severe dementia’. Ratings are guided by functional milestones.
*Staff*		
Sense of Competence in Dementia Care Scale SCIDS [28]	Perceived competence of own delivery of dementia care.	Seventeen statements require a response (varying from ‘not at all’ (1) to ‘very much’ (4)) with reference to the questions ‘how well do you feel you can…’. Scores vary from 17 to 68. A high score indicates a higher level of sense of competence.
Copenhagen Burn-out Inventory CBI [29]	Burn-out	19 Items refer to personal burnout, work-related-burnout and client-related burnout. Scores are averaged over the number of questions for each category. Higher scores indicate higher levels of burn-out.
Cognitive Daisy Questionnaire COG-D Q [15]	Perceived relevance of knowledge about cognitive strengths or weaknesses	Non-validated mixed 3-item scale (open-ended and rating (1–5) items). Each item is analysed separately.

### Participant timeline

**Fig. 3:**
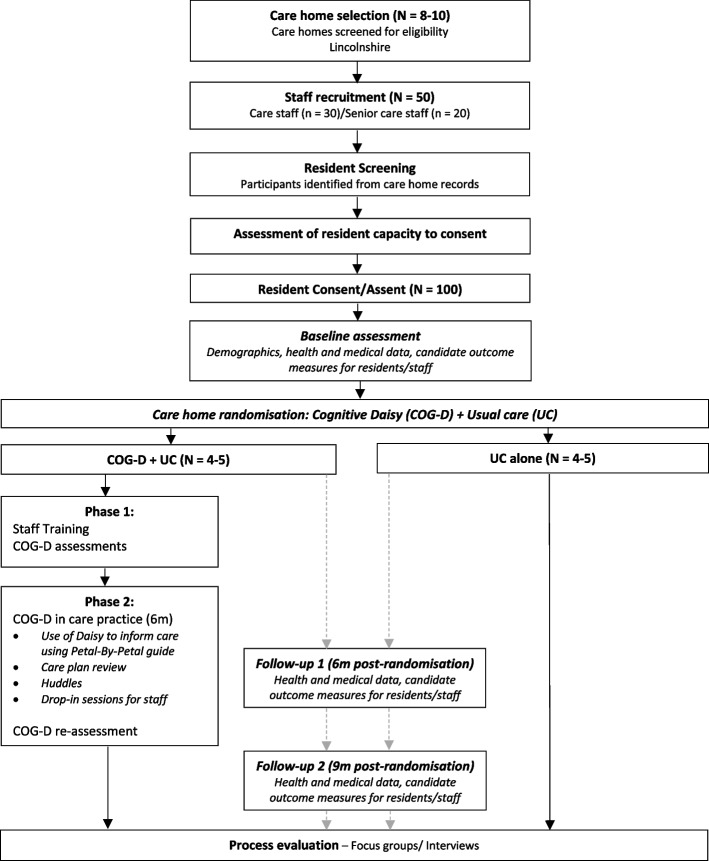
Study flow chart

Care home and individual participant timelines are set out in the study flow-diagram (see Fig. 3):

### Sample size

In line with recommended sample sizes for feasibility studies [18], the aim is to recruit 70 care home residents (35 per arm). To accommodate 30% loss to follow up, 100 residents will be recruited. This sample size will allow for reliable estimation of key feasibility figures, e.g., the 95% confidence interval on retention rate will have the width of ± 9%. To ensure that the target recruitment rate of residents can be achieved from 8 to 10 care homes (it is expected that on average ~ 40–60% of residents will be recruited), only care homes with a minimum of 20 beds will be considered eligible (no maximum limit will be used). We aim to recruit 50 members of staff and will report on the feasibility of recruiting staff for the COG-D intervention.

### Recruitment and consent

Care homes will be recruited in one region in the north-east of England from local care home networks and organizations. Care homes will be screened for initial eligibility via publicly available information. Initially, 8 care homes will be sent recruitment information by email. This will be followed up by a phone/video-conferencing call to confirm interest and to complete initial eligibility screening. A mutually convenient time to visit the care home will then be scheduled to determine full eligibility and complete the recruitment process (i.e., written agreement at the care home level and appropriate management permissions). Additional care homes will be approached if recruitment rates are not achieved with the first 8 care homes.

#### Residents

Anonymous resident screening will take place by the care home manager and a researcher to determine eligibility for approach. An initial discussion about capacity to give informed consent will take place at this point, with the consent process followed for a personal consultee for residents who would be unable to give informed consent to take part in the research. A member of care home staff will then approach eligible residents to ask if they are willing to talk to the researcher. The researcher will explain the study to the resident using the participant information sheet. Consent will be sought for proxy completion of outcome measures, access to their care plan/medical records, and for completion of the COG-D assessments. Resident consent procedures will adhere to the Mental Capacity Act 2005 [19]. Those who can give informed consent will sign or make a mark on the trial consent form. Where a resident is unable to sign, or make a mark, they will be asked to indicate their consent verbally (witnessed by an independent observer). If there is doubt about mental capacity of eligible residents a mental capacity assessment will be completed by the researcher. Where a resident is judged to lack capacity, a personal consultee (usually a family member or friend) will be appointed to provide advice on their wishes. Where no personal consultee is available, a nominated consultee from the care home (a member of staff not involved directly with the study) will be appointed to provide this advice. Personal consultees will be approached in the care home by staff who will introduce the researcher, or by letter/email (sent by the care home manager). In case residents lose capacity during the trial, appropriate guidance on consent in the light of changed capacity will be followed [19], with a personal or nominated consultee appointed to provide advice on the person’s continued participation. At each COG-D assessment session, the resident’s willingness to continue, once initial consent has been secured, will be ascertained.

#### Staff

Short information leaflets about the study will be circulated and placed at convenient locations in the care home. Researchers will approach staff to explain the aim of the study and staff involvement. Interested staff will be provided with the Participant Information Sheet (PIS) and a Consent Form for participation in the study. Researchers will follow up with staff to obtain written consent. A separate consent form is attached to the paper version of the anonymous Staff Questionnaire with candidate outcome measures, and the online version of the Staff Questionnaire is preceded by an eConsent Form.

### Recruitment for interviews/focus groups

#### Residents

Staff and/or the researcher will approach residents they feel may be most able to provide their views on COG-D and its impact (e.g., residents who have resided in the home throughout the study and may be more likely to recall the COG-D assessments). Resident interviews will be brief, using a conversational style and will follow a standardized interview schedule. A sample of up to 2–3 eligible staff will be approached to take part in a focus group and will be provided the PIS and the Consent Form. Relatives or friends eligible to participate in interviews will be identified by the care home manager in discussion with the researcher. Eligible relatives/friends will be approached by the researcher to take part in a focus group held in the care home, at the University of Lincoln, or (in the case of interviews) via an individual telephone/video conferencing interview.

### Assignment of intervention

Randomization of care homes will take place after collection of baseline measures. A web-based randomization system built in the REDCap Cloud electronic data capture system (EDC) will be provided by the Hull Health Trials Unit (HHTU). Randomization by minimization will be undertaken at care home level in 1:1 ratio and stratified by site size (large > 40 beds–small < 40 beds). Each care home will be randomized centrally and dynamically after collection of baseline data is completed. A separate randomization strategy SOP with details on randomization by minimization will be produced and implemented.

To prevent experimenter bias during collection of post-intervention candidate outcome measures (6 and 9 months post-randomization), one of the three trial-specific researchers will be blinded for the condition of each care home (UC or UC + COG-D). Care homes allocated to the COG-D intervention arm will be reminded to remove any references to COG-D (e.g., posters, leaflets, Daisies) in areas where researchers meet care staff who provide proxy data for residents at 6 and 9 months post-randomization. If unblinding happens by accident, then this will be reported to the CI. The feasibility and effectiveness of this approach will be considered in the design of the future planned RCT.

### Withdrawal

Withdrawal may be at an individual participant or care home level and from participation in delivery of the COG-D intervention (where relevant) and/or data collection. All participants will be informed they can withdraw from the study at any point without giving a reason and without it affecting their care/employment. Residents can choose to withdraw from completing the COG-D assessments but may agree to continuation of proxy data collection. Staff may decide to continue using the COG-D in their day-to-day practice if they decide to withdraw from intervention delivery and data collection. If a COG-D assessor does not wish to continue in this role, where feasible a new COG-D assessor will be identified and trained by the research team. Where a relative/friend wishes to withdraw in their role as personal consultee an alternative consultee (nominated consultee, generally a member of staff who knows the resident well) will be sought for ongoing advice.

Residents transferred out of the care home during the trial will not be considered as a withdrawal but termed as lost to follow-up. Researchers will document the date of transfer, and if transferred to a new care home will document the address to confirm if this care home is also taking part in the study. If transferred to another care home participating in the study, the resident’s participation will be reviewed, and relevant consent obtained (i.e., staff proxy informant).

### Data collection methods

As the COG-D intervention has not been tested in a cRCT in care homes to date, this study is designed to address specific areas of uncertainty about conducting a definitive trial of the intervention in this setting. Quantitative outcome data will be collected alongside an integrated mixed-methods process evaluation to explore the feasibility and acceptability of key study parameters and processes from the care home’s and resident’s perspectives.

#### Feasibility outcomes

Recruitment and retention of care homes, residents, and care-staff in both intervention and control conditions will be calculated to inform the number of care homes needed to achieve the target sample size for a future definitive trial. Acceptability, fidelity, and adherence to COG-D intervention protocol will be evaluated with completion rates for staff training and COG-D assessments, use and display of Daisies and revisions to care-plan based on COG-D assessments.

Feasibility of a Health Economic assessment will be explored in the ability to collect the data required (COG-D use, resource use, health and quality of life of residents) to undertake a cost-effectiveness analysis in the definitive trial and estimate potential costs and health benefits of the intervention.

Demographic data for residents (age, ethnicity, length of time in care home, gender, dementia diagnosis) will be collected from care plans. For staff, data will be obtained as part of the anonymous staff questionnaire (further training completed, duration of employment at care home) or in a short questionnaire preceding the staff training (role in care home, ethnicity (to explore diversity), age category, gender).

Candidate outcome measures for residents (by proxy) will be collected in interviews with researchers and staff outcomes are collected in the anonymous staff questionnaire (self-completed). Data completion rates of the candidate outcome measures (at each time point) will be calculated to determine which is the most appropriate primary outcome, agree other study measures and inform sample size calculation for the definitive trial.

#### COG-D assessment scores

(i) Scores from COG-D assessments and re-assessments to evaluate the sensitivity of COG-D to detect cognitive change in residents; (ii) enquiries or comments about cognitive change or requests for re-assessment by care-staff to compare with COG-D assessment results for evaluating the ability of staff to recognize changes in specific cognitive skills; (iii) Concordance rates between COG-D re-assessment scores of researchers and senior care-staff to evaluate ability/potential of staff to conduct assessments in the future CRT. The dementia severity questionnaire (Functional Assessment Staging of dementia (FAST) [20]) will be used to describe the study population and to explore associations between changes in the Cognitive Daisies and assessed dementia severity.

#### Care-plan audit

Conducted pre-post intervention to evaluate if COG-D influences entries in residents’ care-plans. COG-D sheets for care plans accommodate adding resident-specific recommendations for different situations (personal care, mealtimes, scheduled activities). Completion rate and content of these added recommendations will be analyzed. Other sections will be analysed for use of COG-D related terminology (e.g., domain label use to refer to cognitive problems) in relation to suggested changes in care. This analysis will be conducted for all residents for whom consent will have been obtained (whether they complete the COG-D assessment or not) to evaluate transfer of knowledge.

### Process evaluation

Following the MRC guidelines [21] on content of process evaluations, the aim is to explore context, implementation, and mechanisms of impact. Context (i.e., contextual factors and causal mechanisms that affect implementation, intervention mechanisms, outcomes) and mechanisms of impact (i.e., responses to and interactions with the intervention, mediators, unexpected pathways, and consequences) will largely be explored through semi-structured interviews/focus groups after final follow-up with (1) residents who completed the COG-D, (2) care home managers, (3) senior staff who completed the advanced training, (4) care-staff who completed the basic training, (5) relatives of residents who completed the COG-D. Questions will explore experiences of the COG-D implementation, perceptions of efficacy, barriers or facilitators to implementation and suggestions for revisions. Implementation (i.e., process of delivery and what is delivered) will be assessed through (1) audit of COG-D assessments—number of residents invited to complete, number completed initially and at re-assessment (dose, reach) and quality (fidelity), (2) care-plan review of consenting residents—transfer of assessment to care-plan (content, quality, extent), written evidence of implementation (fidelity, adaptations), (3) training records of numbers of staff trained at basic and advanced levels (implementation), (4) interviews/focus groups with care home managers, senior staff and care-staff.

### Data management

HHTU will provide the data systems including a study database built with REDcap cloud EDC, and box file storage system which are both within scope of the HHTU NHS Data Security and Protection Toolkit (Organization Code EE133824-HHTU). Questionnaires can be completed electronically by the researcher or staff directly into REDcap cloud. If paper versions of the questionnaires are used, the responses will be entered into the COG-D data base by a member of the research team. Completed paper versions of the staff questionnaire (PIS and consent form integral) can be sent directly to UoL in self-addressed envelopes or dropped off at the care home in a secure box. Paper copies of data collection forms will be stored in a lockable filing cabinet at the University of Lincoln in a restricted access room and will be identified by a unique study number (study ID) that is assigned when participants consent at baseline. Signed consent forms (including nominated consent) for residents will be stored separately from paper data collection forms as they will contain identifiable information.

Healthcare records of residents will only be accessed by the researcher with consent from the participant (or their consultee). A small amount of identifiable data (e.g., name) will be held to link the resident with their health and social care records (participants with dementia). No directly identifiable personal data of staff will be recorded.

#### Audio recordings

For participants who consent to take part in interviews and focus groups, personal contact details will be stored on the HHTU instance of Box. Interviews will be recorded using an encrypted Dictaphone with the data file uploaded to Box as soon as possible. The file will be deleted from the Dictaphone once the upload is completed. Transcripts will be identified using study ID with other identifying information removed, and only anonymized quotes from interviews and fieldnotes will be used to illustrate themes of analysis.

### Confidentiality

Access to personal data will be limited to authorized members of the research team. All research staff working on COG-D will be required to work to the HHTU Confidentiality Code of Conduct and Confidentiality Audit Procedure. Data will be held in accordance with the General Data Protection Regulation (GDPR 2018). The study will be conducted in compliance with Good Clinical Practice (GCP) and the current approved protocol.

The HHTU EDC system is encrypted in transit and rest, sites can only view and enter data related to their own patients. It offers a robust audit log of data/time/user access and IP is maintained that shows all changes to data records within the system—all access in the system is tracked. Any datasets exported for analysis will be pseudo-anonymized with data identified by study ID.

### Data analysis

Analysis and reporting will adhere to CONSORT 2010 guidelines extension for feasibility trials [22]. A CONSORT flow diagram will be used to display data completeness and resident throughput from eligibility screening, invitation, study acceptance, and final follow-up visit. Feasibility figures on recruitment and retention, COG-D completion, adherence to COG-D implementation protocol, outcome completion rate, and adverse event will be reported and checked against the pre-defined progression criteria.

Signals of efficacy will be analysed in resident and staff candidate outcome measures. Proposed outcome measures (residents: CMAI [23, 24], QUALID [25], BADLS [26], FAST [20], and EQ-5D-5L [27]; care-staff: SCIDS [28], CBI [29], and COG-DQ [15]) will be summarized at baseline, 6-month, and 9-month follow-up. Calculated effect sizes of candidate outcome measures (CMAI, QUALID, and the BADLS), and their corresponding intraclass correlation coefficients (ICCs), will be employed to establish the primary endpoint and sample size for the future definitive cRCT. The calculated completion rates of study measures and questionnaires will be utilized, with their burden and acceptability, to inform the choice of study outcomes for a future large-scale cRCT.

#### Process evaluation

Qualitative data will be analyzed using Framework Method of thematic analysis [30], which provides a systematic model for mapping and managing data. It is ideal for analysis that seeks to reduce large amounts of textual data to analyse by case (care home) and code (area of interest) both within and across cases. It includes seven stages transcription, familiarization, coding (inductive and deductive), developing and analytic framework, applying the analytic framework, charting data into the framework matrix and interpretation.

### Safety monitoring

Adverse and serious adverse events will be recorded and reported in accordance with the HHTU SOP for Adverse Event Reporting for non-CTIMPs and will be in accordance with ICH GCP and the Research Governance Framework 2005. There are no serious adverse events anticipated with participating in this Study. Any adverse events will be recorded in participants’ data collection forms (CRFs) using the adverse event report form and in the care home record.

The care home manager/nominated lead will assess the relationship of each event to the study intervention and classify it as either unrelated or related to the intervention. All adverse events will be recorded and closely monitored until resolution, stabilization, or until it has been shown that the study intervention is not the cause. All AEs will therefore be followed-up until the event has resolved or a decision has been taken for no further follow-up.

As deaths are expected within the trial population, they will not be subject to expedited reporting to the main REC, unless the frequency of deaths observed within the trial population is significantly higher than that expected in the general population. All deaths occurring from the date of consent up to the last data collection visit will be highlighted to the study researcher by the care home (within a week of becoming aware). Death reports will be reviewed by the Chief Investigator monthly and presented at TMG meetings. The Trial Steering Committee (TSC) and Sponsor will be informed of deaths on a regular basis. Reporting of deaths will support avoidance of inappropriate contact by the researchers with the resident’s family following a death. The researcher will contact the care home to ascertain resident status prior to any contact with a relative/friend.

## Discussion

This feasibility study aims to explore the different pathways to impact of COG-D for residents and staff, and to determine to optimal protocol for investigating the effectiveness and cost-effectiveness of COG-D in a future large-scale cRCT in care homes. The inclusion of COG-D re-assessments at the end of the intervention period will provide valuable information about the sensitivity of the Daisies for monitoring change and fluctuations in cognitive functioning of residents with dementia in care homes [7–10]. It will be important to consider these fluctuations in the context of changes in health or medication and to explore the potential combined impact of both factors on independence in daily activities and the optimal way to evaluate this pathway in the future cRCT. Moreover, this information may be valuable for future studies investigating the potential benefits of COG-D in other social care settings and for supporting people with dementia living independently in the community and their family members.

Senior staff who complete the Advanced Training will be invited to lead the COG-D re-assessments with the residents. The proportion of senior staff who choose to accept this role (including facilitators and barriers thereof) will provide important information for further development of the optimal training protocol for the future large-scale RCT. Moreover, feedback from senior staff on the advanced training (in the post-training questionnaire) and their experiences in conducting COG-D assessments (post-trial interviews) will be instrumental in the development of train-the-trainer based training protocol to facilitate larger-scale dissemination of the COG-D package in care homes and other health and social care settings.

### Research ethics and governance

The study was given ethical approval by Wales Research Ethic Committee 1 Cardiff (22/WA/0034); IRAS ID: 305462, Date 21 February 2022. The University of Lincoln are the research governance sponsor. The University of Lincoln will provide indemnity for the protocol and protocol related activity. Individual care homes that will be recruited will be required to provide indemnity for their staff.

### Protocol version and amendments

REC approval was obtained to contact personal consultees by email and to use DocuSign for signatures (approved 26.06.2022). Protocol version 5.

### Dissemination policy

Findings regarding feasibility and signals of efficacy will be submitted to high impact peer-reviewed journals and will be presented at dementia-themed conferences.

## Data Availability

Data sharing plans will be finalized before the end of the study. As a minimum the existence of the dataset and a process for requesting access will be made discoverable.
